# Role of anthraquinones in combating insulin resistance

**DOI:** 10.3389/fphar.2023.1275430

**Published:** 2023-11-20

**Authors:** Wanru Xia, Shuqian Li, LinZehao Li, Shibo Zhang, Xiaolei Wang, Wenyu Ding, Lina Ding, Xiandang Zhang, Zhibin Wang

**Affiliations:** ^1^ Endocrine and Metabolic Diseases Hospital of Shandong First Medical University, Shandong First Medical University and Shandong Academy of Medical Sciences, Jinan, China; ^2^ Shandong First Medical University and Shandong Academy of Medical Sciences, Jinan, China

**Keywords:** anthraquinones, insulin resistance, natural products, intestinal microbiome, antiinflammation

## Abstract

Insulin resistance presents a formidable public health challenge that is intricately linked to the onset and progression of various chronic ailments, including diabetes, cardiovascular disease, hypertension, metabolic syndrome, nonalcoholic fatty liver disease, and cancer. Effectively addressing insulin resistance is paramount in preventing and managing these metabolic disorders. Natural herbal remedies show promise in combating insulin resistance, with anthraquinone extracts garnering attention for their role in enhancing insulin sensitivity and treating diabetes. Anthraquinones are believed to ameliorate insulin resistance through diverse pathways, encompassing activation of the AMP-activated protein kinase (AMPK) signaling pathway, restoration of insulin signal transduction, attenuation of inflammatory pathways, and modulation of gut microbiota. This comprehensive review aims to consolidate the potential anthraquinone compounds that exert beneficial effects on insulin resistance, elucidating the underlying mechanisms responsible for their therapeutic impact. The evidence discussed in this review points toward the potential utilization of anthraquinones as a promising therapeutic strategy to combat insulin resistance and its associated metabolic diseases.

## Graphical Abstract

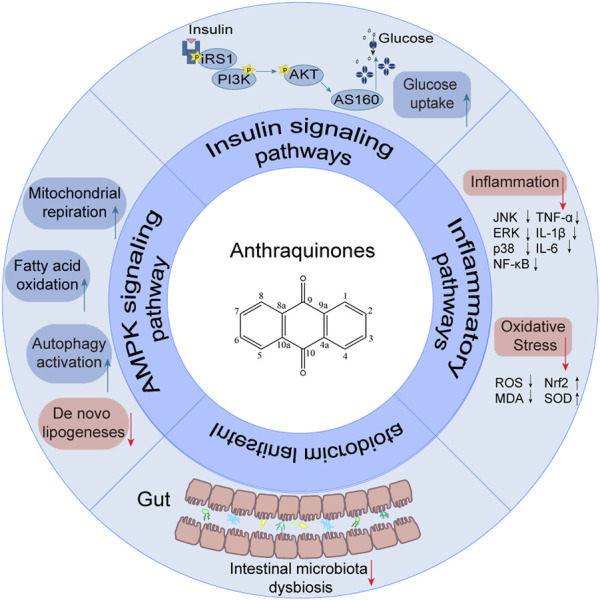

## 1 Introduction

Insulin resistance is characterized by a persistent loss of insulin sensitivity and is a prevalent risk factor contributing to obesity, hypertension, cardiovascular diseases, and type 2 diabetes (James et al., 2021; Wang et al., 2022; Sasaki et al., 2022). Additionally, insulin resistance increases the susceptibility to heart failure and fuels tumor growth, posing a substantial threat to human health and imposing a considerable economic burden on society and families. Notably, its prevalence is on the rise, reaching 20%–40% among young populations in developing countries (Artunc et al., 2016). Consequently, the implementation of effective strategies to ameliorate insulin resistance has become indispensable.

While no medication specifically targets insulin resistance, several antidiabetic drugs, including insulin sensitizers, insulin secretagogues, and alpha-glucosidase inhibitors, have been utilized to improve insulin resistance. However, these treatments often have some adverse effects and limitations. For instance, insulin sensitizers may lead to heart failure (Arnold et al., 2019) and weight gain (Dutta et al., 2023), insulin secretagogues may cause excessive insulin secretion and damage to pancreatic beta cells (Rustenbeck et al., 2004), and alpha-glucosidase inhibitors may result in diarrhea and gastrointestinal discomfort (Taylor et al., 2019).

**FIGURE 1:**
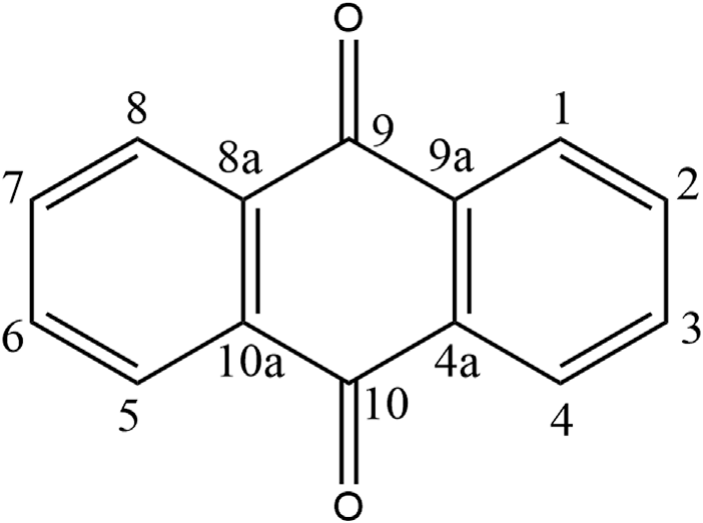
Overview diagram of anthraquinones.

Recently, a plethora of studies have indicated that natural products possessing mild pharmaceutical properties can significantly augment insulin sensitivity, suggesting that natural products may be a new strategy for the treatment of insulin resistance (Zhang et al., 2020; Zhang et al., 2020; Wang et al., 2022; Zhou et al., 2022). These findings underscore the considerable potential of natural products as promising alternatives to conventional treatments for metabolic disorders. Notably, a substantial proportion of these natural products known for their efficacy in combating obesity and ameliorating insulin resistance are rich in anthraquinones (Kumar et al., 2019). For instance, *Cassiae semen* (Ko et al., 2020), *Rheum palmatum L.* (Cui et al., 2019) and *Aloe vera* (Deora et al., 2021), all of which boast anthraquinones, are prominent examples of natural products that are widely employed for the amelioration of metabolic diseases. Anthraquinones, distinguished by their tricyclic diketone pharmacophoric structure (Alam et al., 2019) (Figure 1), constitute a class of plant secondary metabolites. Several studies have substantiated the capacity of anthraquinones to enhance insulin resistance, thereby signifying their promising candidacy as pharmacological agents for mitigating insulin resistance and associated metabolic disorders.

To date, no comprehensive review has been conducted to explore the mechanisms by which anthraquinones ameliorate insulin resistance. This review endeavors to bridge this knowledge gap by providing a systematic assessment of identified anthraquinones and elucidating their respective mechanisms for improving insulin resistance.

## 2 Characteristics of anthraquinones

Anthraquinones (9,10-dioxoanthracenes) are plant secondary metabolites containing a tricyclic dione pharmacophore structure. The anthraquinone ring is the fundamental parent structure of anthraquinones. Anthraquinone monomer refers to chemical compounds containing a single anthraquinone ring, whereas two monomeric anthraquinone units can undergo dehydration and condensation reactions via two distinct pathways to form dimeric anthraquinones (Malik and Müller, 2016). Some studies have revealed that anthraquinones can be substituted with various functional groups, including hydroxyl, alkyl, alkoxy, and sugar units. The specific type, number, and position of substituents on the parent nucleus are critical determinants of natural product bioactivity in this chemical class. For instance, rhein, which has a carboxylic acid group at the sixth substitution position, exhibits significantly greater lipid-lowering activity than aloe-emodin, which has a hydroxyl substitution at the same position (Fang et al., 2022).

Anthraquinones are commonly found in higher plants, such as Polygonaceae, Fabaceae, Rhamnaceae, Rubiaceae, and Liliaceae, either in the form of free anthraquinones or anthraquinone glycosides. Additionally, they are also found in the metabolites of lichens and fungi (Li and Jiang, 2018). It is now well established that anthraquinones exhibit a wide range of biological activities, such as anticancer (Zhang et al., 2021), anti-inflammatory (Xie et al., 2022), antibacterial (Qi et al., 2022), anti-oxidant (Yin et al., 2022), and antiviral effects (Dai et al., 2017). They have also been shown to have great potential in the prevention and treatment of various diseases, including cancer and diabetes.

Emodin, rhein, chrysophanol, aloe-emodin, and physcion are among the most common and representative anthraquinones found in traditional Chinese medicine. These compounds have been identified as the major bioactive components of *R. palmatum L.*, *Polygonum multiflorum*, *Cassiae semen*, *Aloe vera*, and *Senna* (Khurm et al., 2020; Ma et al., 2022). Research has demonstrated the potential of these natural products to improve insulin resistance, making them promising agents for obesity prevention and treatment. *Rumex dentatus L.* is a natural medicinal plant rich in anthraquinones, including emodin. The study demonstrated its significant potential in reducing homeostatic model assessment of insulin resistance (HOMA-IR) and improving insulin resistance in diabetic rats (Elsayed et al., 2020). *Aloe vera* is rich in anthraquinones, including aloe-emodin and aloin. Some studies indicate that these bioactive molecules possess the potential to regulate pancreatic β cell function, suppress fat accumulation, and lower fasting blood glucose (FBG) levels, thus offering an effective therapeutic approach for alleviating obesity (Deora et al., 2021; Fu et al., 2022). Administration of *Aloe vera* extract in obese mice significantly reduced fasting blood glucose levels, improved glucose tolerance, mitigated adipose tissue inflammation, and subsequently ameliorated insulin resistance (Shin et al., 2011; An et al., 2021). A randomized controlled trial with obese individuals demonstrated that *Aloe vera* extracts significantly reduced body weight and HOMA-IR (Zhang et al., 2016). Anthraquinones are also widely distributed among other traditional herbals. *Rheum palmatum L.* is an herbal medicine rich in anthraquinones, including emodin, rhein, and chrysophanol, that has exhibited significant potential in attenuating adipose tissue inflammation and hepatic accumulation of triglycerides in mice. These findings suggest that *R. palmatum L.* may be a potential preventive and therapeutic strategy for obesity (Régnier et al., 2020). Oral administration of *R. palmatum L.* extracts significantly inhibited ectopic fat accumulation and was shown to improve insulin resistance in obese rats (Yang et al., 2016). *Senna*, a natural medicine rich in the anthraquinone-derived natural product sennoside A, has been shown to improve the oxidative stress response and alleviate the inflammatory reaction of adipose tissue, resulting in weight loss in rats (Nayan et al., 2021). Additionally, *Cassiae semen*, a natural medicine rich in anthraquinone-derived natural products such as aurantio-obtusin and alaternin, has been shown to lower FBG and insulin levels and enhance glucose uptake in skeletal muscle in obese mice, thus restoring insulin sensitivity (Wang et al., 2019). Additionally, we have summarized more than ten anthraquinones that can improve insulin resistance through various mechanisms (Table 1).

**TABLE 1:** The Characteristics of Anthraquinones with Improved Insulin Resistance Activity.

Compound	Chemical structure formula	Plant	Overall effect	References
Emodin	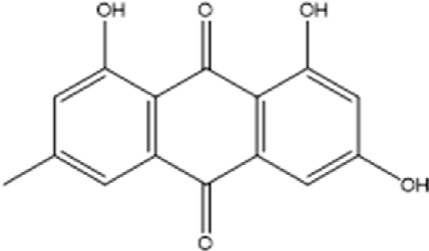	*Rheum palmatum L., Polygonum cuspidatum, Cassiae semen, Senna, Aloe vera*	Reduced fat storage and promoted cellular glucose uptake	Yang et al. (2007), Tzeng et al. (2012a)
Rhein	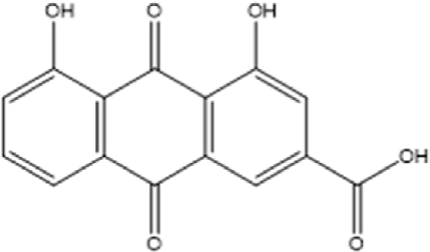	*Rheum palmatum L, Polygonum cuspidatum, Cassiae semen, Senna, Aloe vera*	Reduced inflammatory response	Ji and Gu (2021)
Aloe-emodin	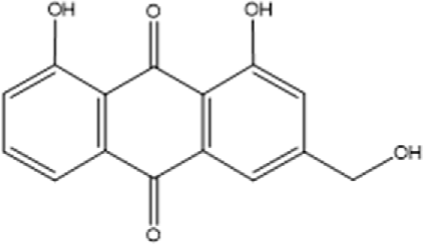	*Rheum palmatum L, Polygonum cuspidatum, Cassiae semen, Senna, Aloe vera*	Reduced inflammatory response	Dou et al. (2019), Quan et al. (2019)
Chrysophanol	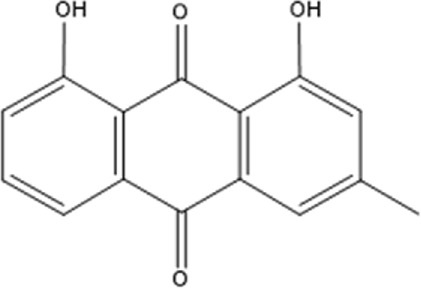	*Rheum palmatum L, Polygonum cuspidatum, Cassiae semen, Aloe vera*	Enhanced cellular glucose uptake and adipose tissue thermogenesis	Liu et al. (2020), Martínez-Aldino et al. (2021)
Physcion	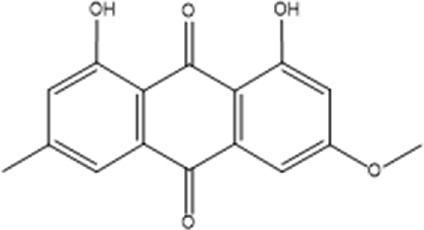	*Rheum palmatum L*	Reduced fat accumulation	Zhao et al. (2017)
Aurantio-obtusin	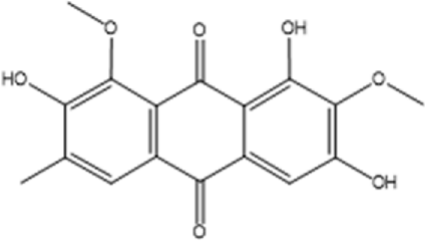	*Cassiae semen*	Reduced adipogenesis; promoted cellular glucose uptake; regulated intestinal flora	Guo et al. (2021), Huo et al. (2022), Li et al. (2022)
Alaternin	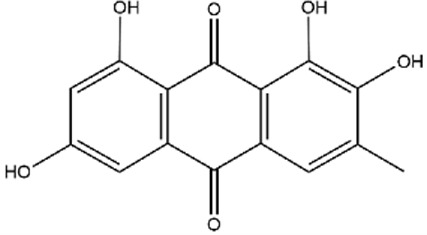	*Cassiae semen, Rhamnus davurica*	Promoted cellular glucose uptake	Jung et al. (2016)
Danthron	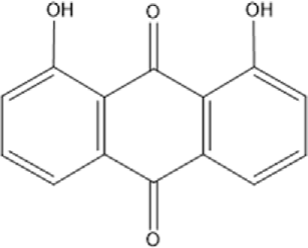	*Rheum palmatum L*	Reduced adipogenesis	(Ma et al., 2021, 2)
Quinalizarin	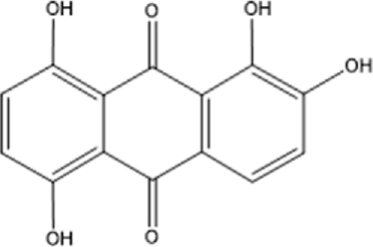	*Rubia cordifolia L*	Reduced adipogenesis	Yang et al. (2016)
Alizarin	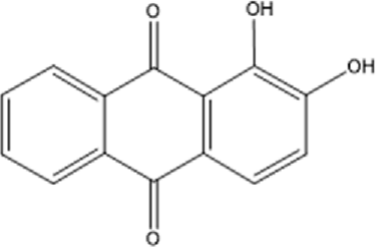	*Rubia cordifolia L*	Promoted cellular glucose uptake	Xu et al. (2019)
Hypericin	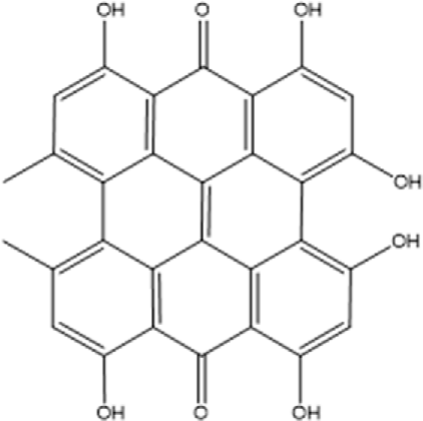	*Hypericum perforatum*	Improved oxidative stress	Liang et al. (2019)
Aloe-emodin-8-O-β-D-glucoside	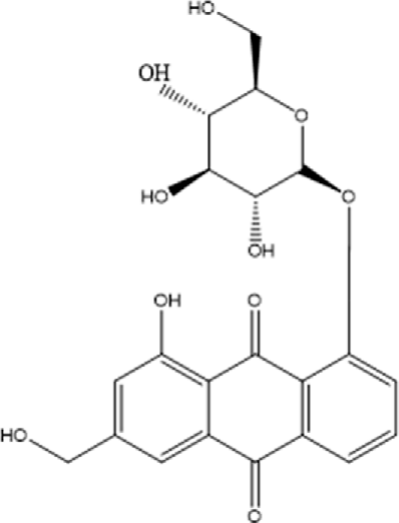	*Cassiae semen*	Promoted cellular glucose uptake	Deora et al. (2021), Fu et al. (2022)
Aloin	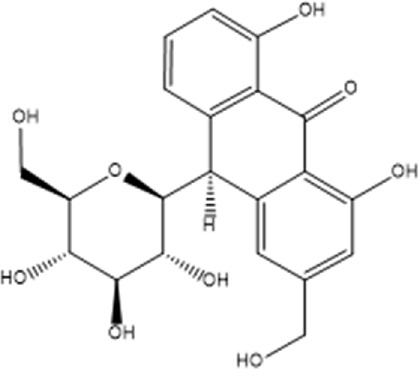	*Aloe vera*	Reduced free fatty acids	Anand et al. (2010)
Sennoside A	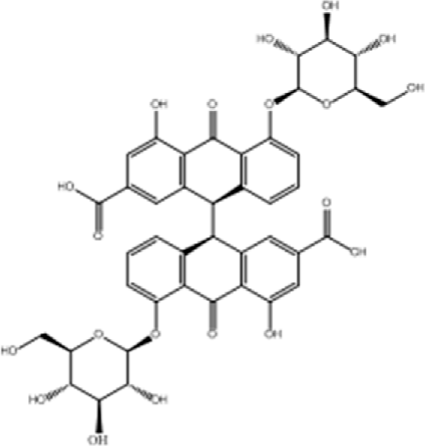	*Senna*	Regulated intestinal flora	Wei et al. (2020)

## 3 Mechanism of insulin resistance improvement by anthraquinones

### 3.1 Anthraquinones improve insulin resistance by attenuating impaired insulin signaling pathways

**FIGURE 2:**
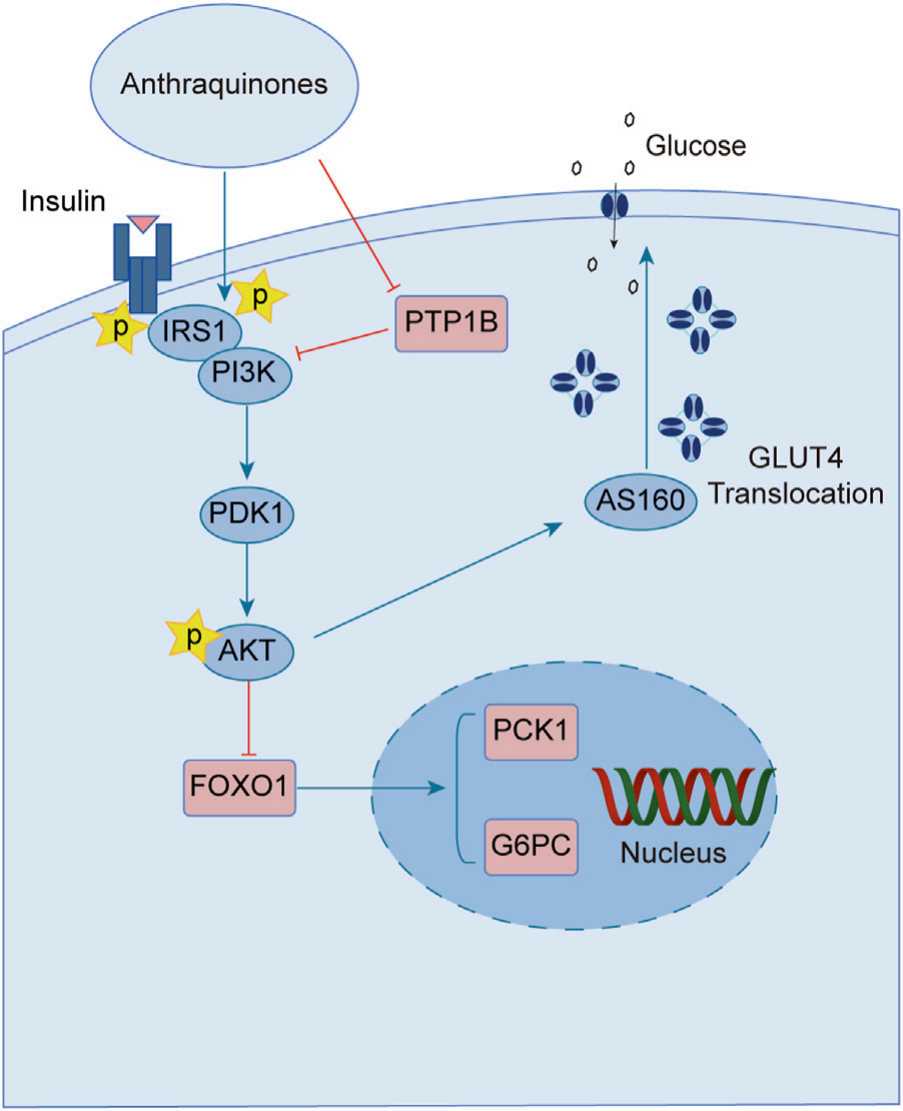
Anthraquinones exert a positive influence on insulin resistance by facilitating the transduction of insulin signaling. These bioactive compounds possess the capability to inhibit the activity of PTP-1B and enhance the expression of PI3K, thereby reinstating the effective conduction of the proximal insulin signaling pathway. Moreover, they elevate the levels of AKT phosphorylation, suppress the expression of FOXO1, and attenuate gluconeogenesis. Furthermore, they promote the expression and translocation of GLUT4, consequently augmenting cellular glucose uptake and fortifying the distal signaling pathways associated with insulin signaling.

Upon engagement with its receptor, insulin sets in motion a myriad of intricate signaling cascades. Insulin resistance manifests when this finely tuned pathway falters, impeding the profound physiological effects of insulin. Remarkably, anthraquinones have demonstrated the ability to alleviate insulin resistance through diverse mechanisms. These include the inhibition of protein tyrosine phosphatase 1B (PTP1B) activity and facilitation of glucose transporter type 4 (GLUT4) expression and translocation, thereby potentially restoring the proper conduction of the insulin signaling pathway (Figure 2).

Insulin binding to its receptor initiates a wide range of signaling cascades that ultimately lead to the uptake and utilization of glucose in insulin target tissues, including skeletal muscles, adipose tissue, and liver. PTP-1B, as a downstream regulatory factor, is a crucial negative regulator of insulin signal transduction. Overexpression of PTP-1B in adipose tissue can lead to dephosphorylation of insulin receptors and inhibit insulin signaling (Venable et al., 2000). Conversely, *PTP-1B* ^*−/-*^ mice show enhanced glucose tolerance and increased systemic insulin sensitivity (Elchebly et al., 1999; Behl et al., 2022), indicating that PTP-1B may serve as a potential therapeutic target for improving insulin resistance. Several studies indicate that anthraquinone compounds could inhibit the activity of PTP1B. Studies have demonstrated that (trans)-emodin-physcion bianthrone and (cis)-emodin-physcion bianthrone isolated from *Polygonum cuspidatum* show potent inhibitory effects against PTP-1B, with corresponding IC_50_ values of 2.77 and 7.29 μM, respectively (Zhao et al., 2017). Furthermore, enzyme kinetic analysis revealed that alaternin extracted from *Cassiae semen* could competitively inhibit PTP-1B activity, with a corresponding inhibition constant (Ki) value of 1.70 μM (Jung et al., 2016). Additionally, molecular docking simulations indicated that the interaction between alaternin and PTP-1B was primarily driven by hydrogen bonding and hydrophobic interactions. A study unveiled the steadfast binding of chrysophanol and emodin to the allosteric site of PTP-1B, shedding light on their intricate association. This site acts as a metastable inhibitor and inactivates the enzyme by blocking the mobility of the catalytic ring of the enzyme (Martínez-Aldino et al., 2021). Anthraquinones have been widely studied, and many of these natural products exhibit inhibitory effects against PTP-1B. Currently, most relevant studies have employed enzyme kinetic analysis or molecular docking simulations to explore their underlying mechanisms. Nevertheless, the cellular and animal realms remain relatively uncharted territories in this domain. Consequently, additional endeavors are imperative to unravel the labyrinthine interplay between anthraquinones and PTP-1B, unmasking their manifold effects across diverse model systems.

GLUT4 is a protein that facilitates the translocation of glucose across cell membranes and is primarily expressed in adipose and muscle tissues. In these tissues, insulin resistance is associated with impaired insulin-dependent translocation of GLUT4, resulting in decreased glucose uptake (Leto and Saltiel, 2012; Zumbaugh et al., 2022). Aloe-emodin-8-O-β-D-glucoside, derived from *Cassiae semen*, exerts its influence by fostering the cellular uptake of glucose through the activation of the phosphatidylinositol pathway and the upregulation of GLUT4 expression (Anand et al., 2010). Upon subjecting insulin-resistant 3T3-L1 cells to the therapeutic influence of emodin, a marked enhancement in cellular glucose uptake was observed. Notably, this effect was found to be partially attenuated by wortmannin, an inhibitor of phosphatidylinositol 3-kinase (PI3K), thereby implicating the PI3K pathway as a crucial route through which emodin stimulates the facilitation of glucose uptake (Yang et al., 2007). The anthraquinone extracts derived from Cassiae semen elicited a substantial elevation in the phosphorylation states of Akt substrate of 160 kDa (AS160), Akt, and PI3K within the skeletal muscle of diabetic rats. This intricate cascade of events fosters the activation of the PI3K-AKT-AS160 signaling pathway, facilitating the translocation of GLUT4 and concomitantly resulting in a commendable reduction in FBG levels as well as fasting serum insulin (FSI) concentrations (Zhang et al., 2018). Aurantio-obtusin propels the activation of the important PI3K-AKT signaling pathway in both hepatic and adipose tissues. This remarkable orchestration induces a discernible reduction in fasting blood glucose levels while elevating glucose tolerance (Guo et al., 2021). Alizarin exerts a profound influence, effectively lowering fasting and postprandial blood glucose levels in diabetic mice. This versatile compound orchestrates a cascade of molecular events, stimulating the phosphorylation of insulin receptor substrate-1 (IRS-1) and Akt proteins while simultaneously enhancing the expression levels of GLUT4. Collectively, these molecular phenomena synergistically contribute to the amelioration of insulin resistance in mice afflicted with diabetes (Xu et al., 2019).

### 3.2 Anthraquinones improve insulin resistance by activating the AMPK signaling pathway

**FIGURE 3:**
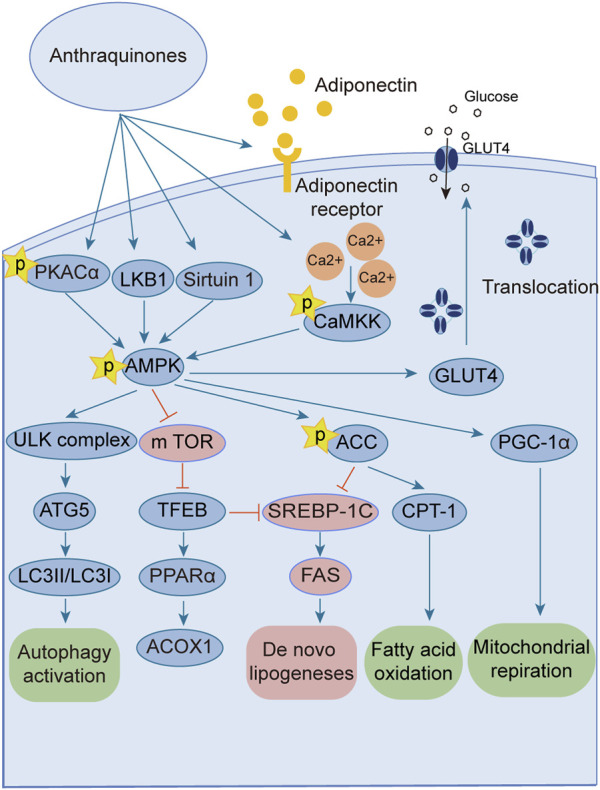
Anthraquinones activate the AMPK signaling pathway through various intricate pathways and mechanisms. This activation subsequently enhances lipid metabolism, culminating in a reduction in lipid synthesis and an increase in fatty acid oxidation. Ultimately, these profound effects contribute to the amelioration of insulin resistance.

The AMPK signaling pathway plays a pivotal role in governing energy metabolism and upholding metabolic equilibrium. Robust evidence supports that AMPK activation enhances insulin sensitivity, promotes glucose uptake, and augments fatty acid oxidation across adipocytes, hepatocytes, and myocytes. Consequently, harnessing the power of AMPK activation has emerged as a potent therapeutic strategy for combating insulin resistance and type 2 diabetes. Notably, several investigations have revealed the ability of anthraquinones, including emodin, aloe emodin, and rhein, to activate the AMPK pathway. These compounds achieve this by increasing the expression and phosphorylation of vital upstream kinases of AMPK, such as protein kinase A (PKA), Ca^2+^/calmodulin-dependent protein kinase kinases (CaMKKs), and adiponectin. Furthermore, these remarkable agents demonstrate the capacity to ameliorate insulin resistance by orchestrating the AMPK pathway, thereby impeding lipid and cholesterol synthesis, enhancing fatty acid oxidation, and fostering glucose uptake (Figure 3).

The AMPK signaling pathway has emerged as a captivating avenue for the prevention and amelioration of insulin resistance (Towler and Hardie, 2007; Lin and Hardie, 2018; Zhang et al., 2022). Positioned as a pivotal kinase regulating energy homeostasis (Diniz et al., 2021), AMPK receives activation signals from an array of influential upstream regulators, including liver kinase B1 (LKB1) (Zhang et al., 2013), CaMKKs (Mungai et al., 2011), transforming growth factor beta-activated kinase1 (TAK1) (Momcilovic et al., 2006), PKA (Hurtado de Llera et al., 2014), and adiponectin (Iwabu et al., 2010; Li et al., 2020). Anthraquinones, including hypericin, danthrone, rhein, and emodin, exhibit the remarkable capacity to augment the expression of key kinases situated upstream of the AMPK signaling pathway, thereby promoting its activation. Through this activation, anthraquinones effectively curtail lipid synthesis, amplify fatty acid oxidation, enhance glucose uptake, and consequently alleviate insulin resistance. Hypericin has garnered attention as a potent agonist of PKA catalytic subunit alpha (PKACα), manifesting its capability to directly bind to PKACα. This direct engagement, in turn, sets forth a cascade of events that activate the PKA/AMPK signaling pathway, thereby effectively impeding the detrimental accumulation of ectopic lipids within the hepatic milieu (Liang et al., 2020). Danthron stimulates the AMPK signaling pathway by augmenting the heterodimerization of retinoid X receptor-alpha (RXRα) and peroxisome proliferator-activated receptor alpha (PPARα) with the adipoR2 promoter (Ma et al., 2021). Rhein exhibits the capacity to increase both the expression and phosphorylation of AMPK protein, thereby effectively activating the AMPK pathway (Lu et al., 2022). Within the composition of *R. palmatum L.*, emodin has been validated as an activator of the AMPK signaling pathway. Its mode of action involves the facilitation of adiponectin expression and the mitigation of oxygen consumption in insulin-resistant C2C12 and 3T3-L1 cells (Chen et al., 2012; Zhang et al., 2015). Moreover, emodin elicits the activation of CaMKK2, a crucial upstream kinase, through the augmentation of intracellular Ca^2+^ concentration within L6 myotubular cells. This consequential event subsequently triggers the activation of the AMPK signaling pathway (Song et al., 2013).

Excessive energy availability promotes heightened flux of free fatty acids (FFAs) and aberrant lipid deposition, thereby contributing to insulin resistance (Nguyen et al., 2005; Jiao et al., 2011). The AMPK signaling pathway assumes a pivotal role in governing lipid metabolism and preventing undue lipid accumulation. Activation of AMPK effectively curtails the activity of acetyl-CoA carboxylase (ACC), alleviating its inhibitory influence on carnitine palmitoyltransferase 1 (CPT-1) and potentiating fatty acid oxidation (Monsénégo et al., 2012; Sheng et al., 2019). Moreover, AMPK activation downregulates the expression of sterol regulatory element-binding protein-1c (SREBP-1c) (Li et al., 2011) and CCAAT enhancer-binding protein alpha (C/EBPα) (Kawaguchi et al., 2002), transcription factors that orchestrate lipogenic gene expression, thereby diminishing lipid synthesis. Therefore, harnessing the AMPK signaling pathway represents an efficacious strategy to counter insulin resistance, as it heightens fat oxidation, suppresses lipogenesis, and fosters lipid homeostasis. Emodin, a naturally occurring compound found in medicinal plants, has demonstrated the capacity to augment fatty acid oxidation through the activation of the AMPK pathway in rats subjected to a high-fat diet (HFD). Its activation of AMPK leads to the upregulation of CPT-1 expression, concomitant with the downregulation of SREBP-1c and fatty acid synthase (FAS) expression, effectively inhibiting lipogenesis and curtailing lipid accumulation. As a result, emodin exerts a beneficial effect on insulin resistance (Tzeng et al., 2012b). Furthermore, intravenous administration of emodin has been observed to stimulate AMPK and ACC phosphorylation in skeletal muscle and liver tissue of HFD-fed mice, leading to reduced fasting blood glucose and fasting insulin levels, as well as improved insulin sensitivity (Song et al., 2013). Chrysophanic acid effectively attenuated weight gain in mice with diet-induced obesity. It also mitigated lipid accumulation and downregulated the expression of adipogenesis-associated factors, such as peroxisome proliferator-activated receptor gamma (PPARγ) and C/EBPα, in 3T3-L1 adipocytes (Lim et al., 2016). In a dose-dependent manner, danthrone exhibited a remarkable capacity to induce the phosphorylation of AMPK and ACC in both HepG2 and C2C12 cells. Furthermore, danthron treatment demonstrated significant efficacy in suppressing lipid synthesis by downregulating the expression of SREBP1c and FAS, thereby leading to reduced levels of total cholesterol (TC) and triglycerides (TGs). Intriguingly, the effects of danthrone on lipid and glucose metabolism were attenuated or reversed when coadministered with the AMPK inhibitor compound C (Zhou et al., 2013). Furthermore, aurantio-obtusin has been shown to induce the phosphorylation of transcription factor EB (TFEB) and bolster autophagic flux within hepatocytes by eliciting AMPK activation. This consequential activation subsequently upregulates the expression of PPARα and acyl-CoA oxidase 1 (ACOX1), thereby stimulating the oxidation of fatty acids. Concurrently, it inhibits the expression of SREBP-1 and FAS, thus culminating in a reduction in lipid synthesis and a decline in the accumulation of lipids in nonadipose tissues (Zhou et al., 2021). TFEB serves as a crucial regulator of autophagy and lysosomal function. Remarkably, TFEB overexpression has been demonstrated to effectively impede weight gain, curtail lipid accumulation, and ameliorate insulin resistance in mouse models of diet-induced obesity (Settembre et al., 2013). The addition of the Physcion supplement increased energy expenditure, contributing to improvements in plasma lipids, adipokines, cytokines, and fecal lipids. Notably, there was a reduction in hepatic FFA synthesis and an increase in FFA oxidation. A significant decrease in lipid synthesis was observed, while lipolysis and oxidation were enhanced.

Activation of the AMPK signaling pathway upholds energy homeostasis by efficiently dissipating surplus energy as heat (Yang et al., 2021). White adipose tissue (WAT) assumes the role of an energy reservoir, storing excess energy in the form of fat. Its accumulation has been closely associated with metabolic disorders (Maqdasy et al., 2022). Conversely, brown adipose tissue (BAT) expends energy through thermogenesis, exerting an inverse relationship with blood glucose levels, insulin resistance, and obesity (Anhê et al., 2019; Xu et al., 2020; Sugimoto et al., 2022; Villarroya and Gavaldà-Navarro, 2022). Certain stimuli, such as cold exposure, exercise, or specific hormonal cues, can instigate a process known as “browning” in WAT (Liu et al., 2022). The process of WAT browning entails the activation of uncoupling protein 1 (UCP-1), a pivotal factor associated with prompt and adaptive thermogenesis (Fedorenko et al., 2012). This process is governed by the transcriptional coactivator peroxisome proliferator-activated receptor-γ co-activator-1α (PGC-1α), which exerts crucial regulatory control over UCP-1 expression and thermogenesis in BAT (Boström et al., 2012). Using primary cultured brown adipocytes as *in vitro* models and HFD-induced obese mice as *in vivo* models, the administration of chrysophanol yielded a notable reduction in weight gain among obese mice. Furthermore, chrysophanol treatment substantially upregulated the expression of UCP1 and PGC1α. Interestingly, in brown adipocytes, the coadministration of Compound C, an inhibitor of AMPK, effectively nullified the impact of chrysophanol on AMPKα, thereby indicating the partial involvement of the AMPKα pathway in chrysophanol efficacy (Lim et al., 2016). Sirtuin 6 (SIRT6) is a multifunctional enzyme with ADP-ribosyltransferase and histone deacetylase activities that orchestrates the recruitment of phosphorylated transcription factor 2 (ATF2), leading to enhanced expression of PGC-1α (Yao et al., 2017). The upregulated expression of SIRT6 increases the phosphorylation of AMPK, thereby ameliorating insulin resistance (Luo et al., 2018; Fan et al., 2019). Chrysophanol administration in mice with HFD-induced obesity substantially elevates SIRT6 and UCP-1 expression within WAT. This finding was further supported by metabolic cage data, elucidating the augmentation of thermogenesis. Importantly, these effects were not observed in Sirt6-deficient mice (Sirt6−/−), underscoring the pivotal role of SIRT6 in mediating the impact of chrysophanol. Collectively, these discoveries highlight the therapeutic potential of chrysophanol in combating obesity and related metabolic disorders by virtue of its ability to upregulate SIRT6, subsequently promoting the upregulation of PGC-1α and UCP-1 in BAT (Liu et al., 2020). Aurantio-obtusin (AO) is a bioactive compound found in Cassiae semen that is a Chinese traditional medicinal herb. It demonstrated remarkable efficacy in enhancing hepatic lipid metabolism in a mouse model of hepatic steatosis. The administration of AO significantly increased mitochondrial metabolism and upregulated UCP1 expression. This effect was achieved through the activation of PPARα signaling, both *in vivo* and in primary brown adipocytes (Yi-Jie et al., 2023).

### 3.3 Anthraquinones ameliorate insulin resistance by inhibiting inflammatory pathways

**FIGURE 4:**
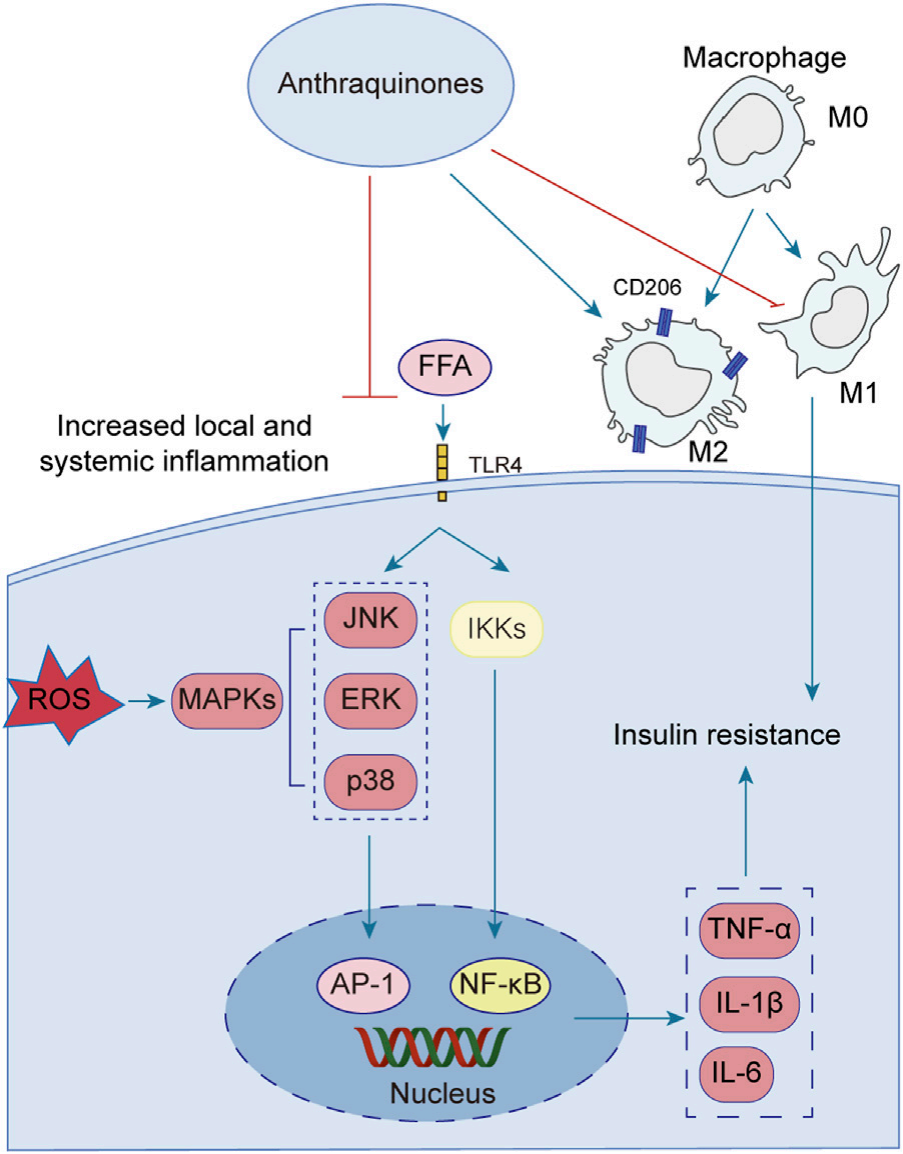
Low-grade chronic systemic inflammation is intricately linked to insulin resistance. Anthraquinones exert a direct inhibitory effect on the MAPK and NF-κB pathways, resulting in the suppression of pro-inflammatory cytokines such as TNF-α, IL-1β, IL-6, and IL-8. Moreover, anthraquinones effectively reduce ROS levels and foster the polarization of M2 macrophages. By mitigating low-grade chronic systemic inflammation through these mechanisms, anthraquinones contribute to the improvement of insulin resistance.

Emerging research has illuminated the pivotal contribution of low-grade chronic inflammation in the development and advancement of insulin resistance. Notably, anthraquinones have demonstrated their potential in mitigating insulin resistance linked to inflammation. These compounds exhibit the ability to ameliorate adipocyte inflammatory infiltration, suppress the secretion of diverse inflammatory factors by adipocytes, induce macrophage M2 polarization, and enhance overall systemic inflammation regulation (Figure 4).

A growing body of evidence highlights the intimate association between low-grade chronic systemic inflammation and insulin resistance (Ahmed et al., 2021). The elevation of FFA triggers the activation of Toll-like receptor 4 (TLR4), initiating a cascade of signaling events involving key regulators such as nuclear factor kappaB (NF-κB) and c-Jun N-terminal kinase (JNK) (Wang et al., 2017; Li et al., 2020; Zhang et al., 2023). Activation of the NF-κB and JNK pathways increases the secretion of various inflammatory mediators, including tumor necrosis factor-alpha (TNF-α), interleukin-1beta (IL-1β), and interleukin-6 (IL-6) (Glass and Olefsky, 2012; Jiang et al., 2022; Park et al., 2022). These pro-inflammatory factors perpetuate systemic inflammation, thus contributing to the persistence of insulin resistance. Several anthraquinones have emerged as potential therapeutics for reducing pro-inflammatory mediators and improving insulin resistance. For instance, chrysophanol demonstrated efficacy by downregulating the expression of TNF-α, IL-1β, IL-6, and IL-8 in mice subjected to a HFD (Lian et al., 2017). Similarly, rhein showcased its beneficial effects by attenuating weight gain, lipid accumulation, and IL-6, IL-1β, and TNF-α levels in adipose tissue and serum of rats with HFD-induced obesity (Ji and Gu, 2021). Molecular docking studies further revealed that rhein effectively bound to TNF-α, IL-6, and NF-κB, with binding energies of −8.9, −7.1, and −7.6 kcal/mol, respectively, suggesting its potential as a modulator of the TNF signaling pathway (Jiang et al., 2022). Additionally, rhein exhibited inhibitory effects on mitogen-activated protein kinase (MAPK) signaling in macrophages, leading to reduced transcription of the proinflammatory mediators TNF-α and IL-1β (Chang et al., 2019). Aloe-emodin demonstrated its capability to diminish TNF-α and IL-6 production while suppressing the NF-κB pathway, thereby restoring insulin signaling and ameliorating insulin resistance (Dou et al., 2019; Quan et al., 2019). Furthermore, emodin was shown to inhibit the expression of adipokines, including TNF-α, IL-1β, and IL-6, in adipocytes, potentially through the inhibition of p38, extracellular-signal-regulated kinase (ERK), and JNK phosphorylation (Fang et al., 2022). In a dose-dependent manner, physcion exhibited the capacity to attenuate the gene expression levels of TNF-α, IL-6, and IL-1β in a hepatocellular carcinoma cell line (HepG2) upon induction of inflammation by lipopolysaccharide (LPS) (Selim et al., 2019). Aloe-emodin significantly attenuated the production of nitric oxide (NO), IL-6, and IL-1β in LPS-stimulated RAW264.7 cells. Western blot analysis revealed that aloe-emodin suppressed the LPS-induced expression of iNOS protein, degradation of IκBα, and phosphorylation of ERK, p38, JNK, and Akt. These findings elucidate the anti-inflammatory properties of aloe-emodin, which likely involve the attenuation of proinflammatory cytokine production in LPS-induced RAW264.7 macrophages through the inhibition of the NF-κB, MAPK, and PI3K signaling pathways (Hu et al., 2014). Furthermore, the nitrogen-containing derivatives of aloe-emodin demonstrated superior efficacy in inhibiting nitric oxide, with an IC_50_ value of 3.15 μM. Furthermore, these derivatives exhibited a significant reduction in the levels of the pro-inflammatory cytokines TNF-α, IL-1β, and IL-6, as well as iNOS and COX-2 enzymes (Shang et al., 2022).

Macrophages can be classified into two distinct subtypes based on their activation state: M1-polarized macrophages and M2-polarized macrophages. In the context of obesity, macrophages tend to favor the M1 phenotype, exacerbating adipose tissue inflammation and contributing to systemic insulin resistance (Li et al., 2018). Conversely, M2 polarization is associated with anti-inflammatory effects, lower body weight, and enhanced insulin sensitivity (Chen et al., 2019). *In vitro* and *in vivo* experiments have demonstrated the inhibitory effects of emodin on the p65-NF-κB complex, along with its ability to enhance the prevalence of M2 (anti-inflammatory)-like phenotype macrophages (Chen et al., 2022). Rhein possesses the ability to shift macrophages toward the M2 phenotype. This was achieved through the downregulation of the M1 marker inducible nitric oxide synthase (iNOS) in mouse colon tissue and the upregulation of CD206, Arg1, IL-10, and ChIL3, which are indicative of M2 macrophage activation (Zhou et al., 2022). The triggering receptor expressed on myeloid cell 2 (TREM2), a member of the immunoglobulin receptor superfamily, has been found to enhance the production of anti-inflammatory cytokines and the expression of M2 marker genes when overexpressed (Korvatska et al., 2020). Upregulation of TREM2 has been demonstrated to mitigate insulin resistance induced by obesity (Carrasco et al., 2019). In mice with HFD-induced obesity, emodin effectively induced the polarization of M2 macrophages through the upregulation of TREM2 expression. This intervention notably alleviated local and systemic inflammation, curbed weight gain and lipid accumulation, reduced fasting glucose and fasting insulin levels, and improved insulin sensitivity (Yu et al., 2021). Rhein exhibited notable efficacy in reducing tissue inflammation and facilitating the transition of macrophages toward an M2 polarization state in an LPS-induced model. *In vitro* experiments demonstrated that rhein effectively mitigated intracellular ROS levels, suppressed the activation of P65, and thereby hindered macrophage polarization toward an M1 phenotype. Mechanistically, the protective effects of rhein were attributed to its modulation of the nuclear factor of activated T cells c1 (NFATc1)/TREM2 axis, as evidenced by the substantial attenuation observed in blocking experiments targeting both TREM2 and NFATc1 (Li et al., 2023).

The interplay between oxidative stress and inflammation can mutually aggravate insulin resistance. Reactive oxygen species (ROS), such as superoxide dismutase (SOD) and malondialdehyde (MDA), can stimulate the production of inflammatory factors, while cellular inflammatory factors, in turn, promote the generation of free radicals (Kwak et al., 2017). Oxidative stress poses detrimental effects on pancreatic beta cell function, leading to apoptosis and exacerbating insulin resistance (Costes et al., 2006). Notably, aloe-emodin has demonstrated the ability to reduce ROS levels in RIN-5F cells exposed to high glucose conditions, thus safeguarding these cells (Alshatwi and Subash-Babu, 2016). Physcion, a bioactive compound derived from rhubarb, exhibits notable properties, such as antihypertensive, antibacterial, and antitumor activities. Remarkably, physcion demonstrated the capacity to reduce body weight and plasma TG levels in rats subjected to a HFD. Palmitic acid increased the levels of ROS and MDA and reduced the levels of NO, SOD and GSH-Px. These trends were reversed by physcion. In addition, physcion reversed PA-induced activation of the NF-κB/TNF-α pathway in HUVECs (Wang et al., 2023). The pancreatic and duodenal homeobox-1 (PDX1) protein plays a pivotal role in pancreatic development, maturation, and the functioning of β cells (McKinnon and Docherty, 2001). In the context of glycotoxicity and lipotoxicity, oxidative stress further hinders PDX1 expression, resulting in β cell dysfunction and apoptosis (Hong et al., 2012). Conversely, hypericin enhances PDX1 expression through ERK activation in mice subjected to high-fat and high-glucose diets, thereby ameliorating glucose intolerance and insulin resistance. This intervention also leads to reduced fasting blood glucose levels, attenuation of islet-β cell apoptosis, and inhibition of nitric oxide (NO) production induced by glucotoxicity and lipotoxicity (Liang et al., 2019). Furthermore, *in vivo* experimentation revealed that emodin impeded the manifestation of TNF-α, IL-6, and MDA within both the circulating serum and tissues while concurrently augmenting the concentrations of SOD and GSH (Shang et al., 2021). Rhein exhibited a potent inhibitory effect on LPS-induced intestinal inflammation and oxidative stress. This was evidenced by a significant reduction in serum and intestinal levels of TNF-α, IL-1β, IL-6, and nitric oxide. Additionally, it downregulated MDA levels in the small intestine. Remarkably, rhein also inhibited the phosphorylation of JNK and p38 MAPK while activating the nuclear factor E2-related factor 2 (Nrf2) pathway (Zhuang et al., 2019).

### 3.4 Anthraquinones mitigate insulin resistance by regulating the intestinal microbiota

**FIGURE 5:**
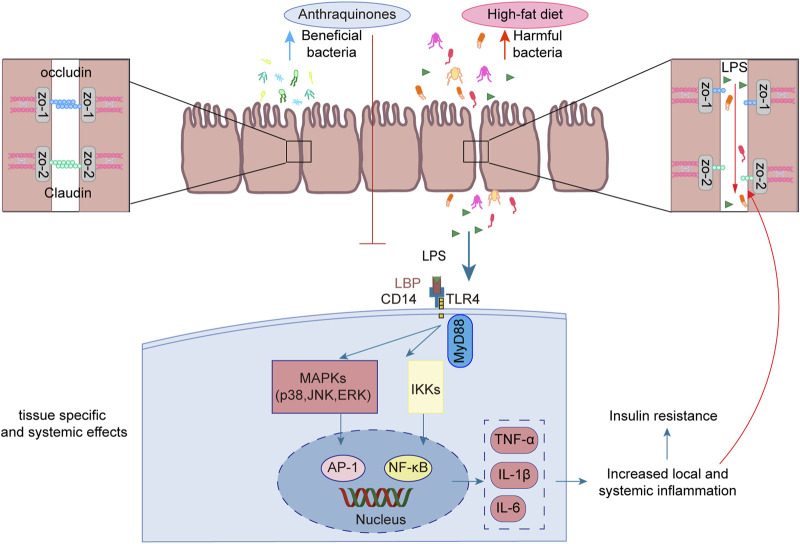
Anthraquinones efficiently attenuated the elevation in plasma LPS levels induced by a HFD while simultaneously dampening LPS-induced inflammatory responses in intestinal epithelial cells. Furthermore, these compounds demonstrated the ability to safeguard the continuity and integrity of colonic enterocytes through the upregulation of crucial tight junction proteins, including occludin, claudin, and ZO-1.

Emerging studies have unveiled the close interconnection between metabolic disorders and the perturbations observed in the composition and functionality of the intestinal microbiota (Ussar et al., 2015; Zeng et al., 2020). Manipulating the gut microbiota has emerged as a promising therapeutic strategy to enhance insulin sensitivity in the host (Chambers et al., 2019; Naderpoor et al., 2019). Anthraquinones have been demonstrated to effectively modulate gut dysbiosis by promoting the proliferation of beneficial bacteria while concurrently suppressing the abundance of potentially pathogenic counterparts. Notably, these compounds have been shown to enhance insulin resistance through the preservation of gut mucosal integrity and the reduction in metabolic endotoxemia. As such, anthraquinones present a promising approach for restoring the gut microbiota (Figure 5).

LPS, also referred to as endotoxins, is a sizable compound present in the outer membrane of Gram-negative bacteria (Simpson and Trent, 2019). Following the destruction of Gram-negative bacteria, the breakdown of their cell walls results in the release of LPS into the surrounding milieu (Tulkens et al., 2020). High-energy diets have been demonstrated to elicit heightened plasma LPS levels in murine experiments, as well as in a substantial cohort of healthy men drawn from a population-based sample (Amar et al., 2008). The translocation of LPS across the gut lining serves as a hallmark associated with insulin resistance, obesity, and diabetes. The leakage of LPS into the bloodstream initiates a state of low-grade inflammation, exerting profound effects on the liver, adipose tissue, and muscle metabolism (Cani et al., 2008). Long-term subcutaneous infusion of LPS in mice results in various alterations, including elevated weight gain, insulin resistance, WAT inflammation, heightened systemic LPS levels, and increased intestinal permeability (Cani et al., 2007). The regulation of tight junction permeability plays a pivotal role in maintaining the integrity of the intestinal barrier. Disruption of these tight junctions results in compromised barrier function, leading to “leakage” and subsequently causing an elevation in intestinal permeability (Ma et al., 2004). Additionally, an increase in LPS leads to its binding with TLR4, consequently activating the TLR4 signaling pathway. This signaling cascade involves downstream regulators such as NF-κB and JNK (Płóciennikowska et al., 2015). Rhein demonstrated a salutary influence by promoting body weight reduction and enhancing glucose tolerance in mice with diet-induced obesity. Moreover, it efficiently attenuated the surge in plasma LPS levels induced by a HFD while concurrently mitigating the accumulation of proinflammatory macrophages within the colon (Wang et al., 2016). Another investigation demonstrated the ameliorative effects of rhein on LPS-induced intestinal barrier injury, achieved through the modulation of Nrf2 and MAPK signaling pathways (Zhuang et al., 2019). Rhein restores the expression of claudin-1, E-cadherin, and mucus secretion to reduce intestinal permeability in chronic mouse colitis model induced by dextran sulfate sodium (Wu et al., 2020). Emodin demonstrated the capacity to suppress LPS-induced inflammatory responses in intestinal epithelial cells while simultaneously enhancing intestinal barrier function through the upregulation of ZO-1 and occludin expression (Zhang et al., 2020). Aurantio-obtusin exerts a favorable influence on intestinal barrier function by upregulating the expression of occludin and ZO-1 in HFD-fed mice. Moreover, it reduces serum LPS levels and attenuates the production of inflammatory mediators (Luo et al., 2021). Sennoside A was observed to safeguard the continuity and integrity of colonic enterocytes in mice with diet-induced obesity by upregulating the expression of tight junction proteins, namely, occludin, claudin-2, and ZO-1. This mechanism effectively restores colonic barrier function (Ma et al., 2020).

In murine models, it has been demonstrated that the consumption of a HFD induces notable modifications in the composition of the gut microbiota (Rabot et al., 2016; Tan et al., 2021). This perturbation is characterized by a decline in the abundance of beneficial bacteria, such as *Bifidobacterium* (Yang et al., 2022) and *Lactobacillus* (Lee et al., 2021), coupled with an elevation in the prevalence of potentially pathogenic microbes, including *Bilophila wadsworthia* (Natividad et al., 2018) and *Ruminococcus gnavus* (Grahnemo et al., 2022). The intervention of anthraquinones of Cassiae semen effectively increased *Bacteroides*, *Lactobacillus*, and *Parabacteroides* in HFD-fed rats (Mei et al., 2015). Another investigation demonstrated a significant abundance of *Lactobacillus* after rhein treatment (Wu et al., 2020). The modulation of gut microbiota contributed to the amelioration of metabolic syndrome in mice subjected to a HFD (Shi et al., 2019; Wang et al., 2020). In a double-blind randomized placebo-controlled pilot trial utilizing oral fecal microbiota transplantation (FMT) capsules, patients receiving FMT exhibited enduring alterations in their microbiomes linked to obesity, converging toward those characteristic of the lean donor (*p < 0.001*) (Allegretti et al., 2020). An increase in the abundance of potentially advantageous bacteria may ameliorate insulin resistance. *Akkermansia muciniphila* has been identified as a key regulator of energy metabolism, glucose tolerance, and the maturation and functionality of the immune system in human individuals (Yoon et al., 2021). Furthermore, the absence of *A. muciniphila* has been implicated in the disruption of gut barrier integrity, exerting consequential effects on other bacterial populations, ultimately precipitating the development of insulin resistance (Yang et al., 2019). A study demonstrated that aurantio-obtusin significantly augments the abundance of *Akkermansia* winderi in mice fed a HFD (Luo et al., 2021). Rhein effectively restrained the elevated plasma LPS levels induced by a HFD and modulated the gut microbiota by reducing *Bacteroides-Prevotella* spp. and *Desulfovibrio* spp. DNA while simultaneously increasing *Bifidobacterium* spp. and *Lactobacillus* spp. DNA (Wang et al., 2016).

## 4 Conclusion and future perspectives

In recent years, metabolic disorders have emerged as a pressing global health concern. Projections suggest that obesity will affect approximately one billion individuals by 2030, and diabetes cases will escalate to 783 million by 2045, posing significant challenges to healthcare systems worldwide. Numerous studies have indicated that anthraquinones hold promise in improving insulin resistance, a pivotal factor in preventing and treating various diseases, including diabetes, obesity, and other metabolic syndromes. Thus, this systematic review aims to comprehensively elucidate the mechanisms underlying the potential of anthraquinones in ameliorating insulin resistance, thereby fostering a deeper understanding of their therapeutic applications.

While numerous anthraquinones exhibit potential in ameliorating insulin resistance, the current research predominantly focuses on key compounds such as emodin, chrysophanol, rhein, and aloe-emodin. The precise mechanisms by which these compounds improve insulin resistance warrant further investigation. Moreover, existing studies primarily utilize animal models, cell culture models, or enzymatic methods to explore the potential of anthraquinone natural products in insulin resistance improvement. Therefore, well-designed, multicenter trials with large sample sizes are imperative to evaluate the effects of anthraquinones in human subjects with insulin resistance. It is hoped that upon integrating anthraquinones as treatment options for insulin resistance, they will prove to be both safer and more efficacious, offering innovative approaches to addressing metabolic disorders.

Overall, the potential of anthraquinones to improve insulin resistance through multiple pathways makes them a promising candidate for the treatment of insulin resistance and related metabolic disorders.

## Figures and Tables

**Table:** 

**ACC**	Acetyl-CoA carboxylase
**ACOX1**	Acyl-CoA oxidase 1
**AMPK**	AMP-activated protein kinase
**AS160**	Akt substrate of 160 kDa
**ATF2**	Activating transcription factor 2
**BAT**	Brown adipose tissue
**CaMKKs**	Ca2+/calmodulin-dependent protein kinase kinases
**C/EBPα**	CCAAT enhancer-binding protein alpha
**CPT-1**	Carnitine palmitoyltransferase 1
**ERK**	Extracellular-signal-regulated kinase
**FAS**	Fatty acid synthase
**FBG**	Fasting blood glucose
**FFA**	Free fatty acids
**FSI**	Fasting serum insulin
**GLUT4**	Glucose transporter type 4
**HFD**	High-fat diet
**HOMA-IR**	Homeostatic model assessment of insulin resistance
**IL-1β**	Interleukin-1beta
**IL-6**	Interleukin-6
**iNOS**	Inducible nitric oxide synthase
**IR**	Insulin resistance
**IRS-1**	Insulin receptor substrate-1
**JNK**	C-Jun N-terminal kinase
**LKB1**	Liver kinase B1
**LPS**	Lipopolysaccharide
**MAPK**	Mitogen-activated protein kinases
**MDA**	Malondialdehyde
**Mn-SOD**	Manganese superoxide dismutase
**NF-κB**	Nuclear factor kappaB
**NO**	Nitric oxide
**PDX1**	Pancreatic and duodenal homeobox 1
**PGC-1α**	Peroxisome proliferator-activated receptor-γ co-activator-1α
**PI3K**	Phosphatidylinositol 3-kinase
**PKA**	Protein kinase A
**PPARα**	Peroxisome proliferator-activated receptor alpha
**PTP1B**	Protein tyrosine phosphatase 1B
**ROS**	Reactive oxygen species
**RXRα**	Retinoid X receptor-alpha
**SIRT6**	Sirtuin 6
**SOD**	Superoxide dismutase
**SREBP-1c**	Sterol regulatory element-binding protein-1c
**TAK1**	Transforming growth factor beta-activated kinase1
**TFEB**	Transcription factor EB
**TLR4**	Toll-like receptor 4
**TNF-α**	Tumor necrosis factor-alpha
**TREM2**	Triggering receptor expressed on myeloid cell 2
**UCP-1**	Uncoupling protein 1
**WAT**	White adipose tissue
**ZO-1**	Zona occludens-1
